# 应用FR靶向PCR法检测CTC在肺癌诊断中的临床价值：初步研究

**DOI:** 10.3779/j.issn.1009-3419.2016.12.03

**Published:** 2016-12-20

**Authors:** 欢欢 连, 志丹 丁, 东风 袁, 杰 马, 建军 秦

**Affiliations:** 1 450008 郑州，河南省肿瘤医院胸外科 Department of Thoracic Surgery, Henan Oncology Hospital, Zhengzhou 450008, China; 2 450008 郑州，河南省肿瘤医院分子病理科 Department of Molecular Pathology Center, Henan Oncology Hospital, Zhengzhou 450008, China

**Keywords:** 叶酸受体, 循环肿瘤细胞, 靶向PCR, 辅助诊断, 术后监测, Folate receptor, circulating tumor cell, Targeted PCR, Auxiliary diagnosis, Postoperative monitor

## Abstract

**背景与目的:**

评价一种通过叶酸受体(folate receptor, FR)检测循环肿瘤细胞(circulating tumor cell, CTC)的方法用于肺癌临床诊断的实用性和可行性及进一步探究CTC在肺癌术后复发的预测价值。

**方法:**

通过免疫磁珠负向富集方法从3 mL外周血中捕获循环肿瘤细胞，再用肿瘤特异性叶酸配体-寡核苷酸偶和物标记捕获的循环肿瘤细胞，洗去没有结合的偶和物后，洗脱下特异性结合的偶合物的寡核苷酸用于定量PCR扩增分析。

**结果:**

97例肺癌患者的CTC水平高于肺部良性疾病患者(*P* < 0.001)。本检测方法以8.7 Folate Units/3 mL为cutoff值，结果显示靶向PCR法对肺癌的检测灵敏度为82.5%，特异性为72.2%，特别是在Ⅰ期肺癌灵敏度达到86.8%。与其他肿瘤标志物(NSE、CEA、CYFRA21-1)比较，CTC对肺癌及Ⅰ期肺癌具有较高的诊断准确性(0.859; 95%CI: 0.779-0.939)和(0.912; 95%CI: 0.829-0.994)。5例肺癌患者术后2周内CTC水平高于cutoff值。

**结论:**

叶酸受体阳性循环肿瘤细胞可以应用于肺癌的临床诊断，即使是对早期非小细胞肺癌(non-small cell lung cancer, NSCLC)的诊断。

## Abstract (en)

**Background and objective:**

The aim of this study is to determine the efficacy and feasibility of a novel folate receptor (FR)-based circulating tumor cell (CTC) detection method in the diagnosis of lung cancer. CTCs were collected from 3 mL of blood based on negative enrichment by immunomagnetic beads and then labeled by a conjugate of a tumor-specific ligand folate and an oligonucleotide.

**Methods:**

After washing off redundant conjugates, the bound conjugates were removed and analyzed by quantitative polymerase chain reaction.

**Results:**

The CTC levels of 97 patients with lung cancer were significantly higher than that of the controls (18 patients with benign lung diseases; *P* < 0.001). With a threshold of 8.7 Folate units, the method showed a sensitivity of 82.5% and a specificity of 72.2% in the diagnosis of lung cancer, especially a sensitivity of 86.8% in stage Ⅰ disease. Compared with the existing clinical biomarkers such as neuron-specific enolase (NSE), carcinoembryonic antigen (CEA), and CYFRA21-1, the method showed the highest diagnostic efficiency in lung cancer (area under the curve, 0.859; 95%CI: 0.779-0.939) and stage Ⅰ lung cancer (area under the curve, 0.912; 95%CI: 0.829-0.994). For future work, the CTC levels of 5 lung cancer patients higher than 8.7 Folate units/3 mL in their postoperation.

**Conclusions:**

FR-positive CTCs were feasible diagnostic biomarkers in patients with lung cancer, as well as in early-stage tumors.

肺癌是目前全球发病率最高的恶性肿瘤之一，并且在我国的发病率和死亡率高居榜首。非小细胞肺癌(non-small cell lung cancer, NSCLC)占肺癌总数的80%，是最为常见的肺癌，主要包括腺癌、鳞状细胞癌即肺鳞癌、大细胞未分化癌三类。NSCLC的治疗要根据肺癌的临床分期来进行。对Ⅰ期、Ⅱ期、Ⅲa期主要以手术切除为主，淋巴转移显著者于手术前可辅以化疗或放疗。经手术后，Ⅰ期患者5年生存率约70%，Ⅱ期5年生存率约50%，Ⅲ期能做手术或不能手术化放疗联合治疗者5年生存率15%-30%^[1, 2]^。患者致死的主要原因是NSCLC的复发和转移^[3]^。肿瘤细胞进入血液循环是肿瘤发生远处转移的关键步骤之一^[4]^，因此对NSCLC患者循环肿瘤细胞(circulating tumor cell, CTC)检测的研究已受到越来越多的重视。

CTC检测将有助于NSCLC的早期诊断^[5-7]^、复发转移监测^[8, 9]^、判断患者预后和化疗疗效评估^[10]^。与淋巴结、骨髓相比，外周血标本易获得、创伤性小、可反复采集，是临床上常规检测较为理想的标本来源。由于上皮间质转化(epithelial-to-mesenchymal transition, EMT)，目前利用CTC上皮表型(EpCAM)检测恶性肿瘤患者CTC的方法有局限性^[11]^。NSCLC患者的CTC经常不表达上皮细胞表型^[12]^。一个前瞻性的研究^[13]^显示只有32%的远处转移的NSCLC患者(19/60)CTC计数大于CellSesrch检测系统的基线值。

在一些癌症中叶酸受体(folate receptors, FRs)表达量很高，特别是在卵巢癌和肺癌中，然而在正常组织中极少存在^[14]^。因此，FR可以作为一个用于肺癌患者CTC的检测靶点。已有文献^[15]^报道，基于FR靶向PCR CTC检测方法的诊断肺癌的敏感度80%，特异性为88%。该方法尤其对Ⅰ期NSCLC患者的诊断灵敏度达到67.2%^[6]^。

由于CTC可以评估肿瘤的发生发展状态^[16]^，而且手术切除原发灶后，血液中CTC数量应该为阴性。因此，理论上而言，在术后监测过程中，CTC数量阳性，可能提示肿瘤存在复发的可能性。目前仅有少数研究在这方面进行了探索，Sawabata等^[8]^对9例接受肺叶切除术的NSCLC患者进行了术前术后的CTC检测。结果发现1例患者术前在外周血中检出CTC，而有3例患者在手术后即刻检测中在外周血中发现了CTC，所有患者在术后10天以后均无法检出CTC。对于CTC在复发监测中的作用，仍需更多研究来探索。

本研究的目的是验证通过FR靶向PCR检测CTC的方法用于肺癌特别是早期患者诊断的有效性和可行性及进一步探究CTC在肺癌术后复发的预测价值。

## 1 材料与方法

### 1.1 样本入组

2015年1月-2016年5月，研究共入组了97例即将接受治疗的肺癌患者(包括但不限于肺鳞癌与肺腺癌)。入组样本由河南省肿瘤医院胸外科提供。所有患者已经细胞学或组织病理学确诊。在研究之前，患者均未接受抗肿瘤治疗或患有其他的肿瘤史。肿瘤分期根据第七版美国癌症联合委员会关于癌症分期指南。此外，入组18例经影像学或组织病理学确诊为肺部良性疾病的患者。患者特征见表 1。

**1:** 患者特征 Characteristics of lung cancer and benign diseases

Characteristics	Number	Ratio
Lung cancer (*n*=97)		
Age, years (Median, range)	61 (38-72)	
Gender (Male/Female)	64/33	
Smoking	59	60.8%
T stage		
T1	24	24.7%
T2	53	54.6%
T3	12	12.4%
T4	6	6.2%
Tx	2	2%
N stage		
N0	51	52.6%
N1	12	12.4%
N2	16	16.5%
N3	13	13.4%
Nx	5	5.2%
M stage		
M0	84	86.6%
M1	13	13.4%
Grade		
Well differentiated (G1)	3	3.1%
Moderately differentiated (G2)	72	74.2%
Poorly differentiated (G3)	22	22.7%
Tumor stage		
Ⅰ	38	39.2%
Ⅱ	17	17.5%
Ⅲ	26	26.8%
Ⅳ	13	13.4%
Unsureness	3	3%
Histopathologic subtype		
ADC	50	51.5%
SCC	36	37.1%
SCLC	9	9.3%
Other	2	2.1%
Benign diseases (*n*=18)		
Age, years (Median, range)	51 (37-66)	
Gender (Male/Female)	8/10	
Smoking	5	27.8%
ADC: adenocarcinoma; SCC: squamous cell carcinoma; SCLC: small cell lung cancer.		

### 1.2 血液样本处理

抽取4 mL外周血样本，采用6 mL抗EDTA采血管(BD Diagnostics, Sparks, MD)收集。样本储存在4 ℃，并且在24 h内完成血液处理。血液样本取自即将做手术治疗患者的肘前静脉血。CTC检测人员不知道检测样本的分组(单盲)。在检测人员获得样本的时候，所有样本已经被“去标识”了。

### 1.3 CTC检测

CytoploRare^®^叶酸受体阳性循环肿瘤细胞检测试剂盒由格诺思博生物科技(上海)有限公司提供。试剂盒的原理如前所述^[5-7]^，且经过改良用于肺癌患者的CTC检测。简单说，方法由两部分组成：①免疫磁珠法负向富集叶酸受体阳性循环肿瘤细胞；②通过配体-靶向PCR定量检测CTC。引物序列为：反转录(RT)引物(一段寡核苷酸用来偶联肿瘤特异性叶酸配体)，5′-CTCAACTGGTGTCGTGGAGTCGGCAATTCAGTTGAGGGTTCTAA-3′；前引物，5′-TATGATTATGAGGCATGA-3′；后引物，5′-GGTGTCGTGGAGTCG-3′；TaqMan探针，5′-FAM-CAGTTGAGGGTTC-MGB-3′^[17]^(图 1)。

**1:**
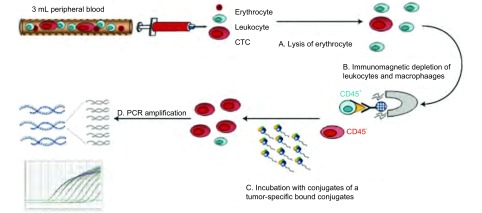
CytoploRare叶酸受体阳性循环肿瘤细胞检测试剂盒简易流程图 The Flowchart of cytoplorare folate receptor-positive cell detection kit. CTC: circulating tumor cell.

根据试剂盒说明书，3 mL全血样本中加入红细胞裂解液(体积:体积=1:4)，在4 ℃裂解15 min，去除红细胞；然后，加入150 μL抗CD45磁珠和50 μL抗CD14磁珠，4 ℃孵育30 min，分别去除白细胞和巨噬细胞。之后，富集的CTC加入10 μL含有肿瘤特异性叶酸配体-寡核苷酸偶合物的探针标记液，室温孵育40 min。接着，这些样品加入1 mL洗涤缓冲液，4 ℃，500 *g*，离心10 min，重复三次，去除未结合的探针。最后，加入120 μL洗脱缓冲液，4 ℃孵育2 min，洗脱下已结合的探针，离心收集，加入24 μL中和缓冲液，用于荧光定量PCR扩增分析。在PCR分析阶段，PCR信号与数据的收集都是通过ABI7300(Life Technologies, Carlsbad, CA)完成。ABI7300仪器反应条件为：95 ℃变性2 min，40 ℃退火30 s，72 ℃延伸30 s，8 ℃冷却5 min；40个循环，95 ℃变性10 s，35 ℃退火30 s，72 ℃延伸10 s^[18]^。

研究中，我们设定一个自我定义的测量单位“Folate Unit”，即3 mL血液中检测到的CTC水平。如果3 mL血液中检测到1个CTC，就定义为1 Folate Unit。含有寡核苷酸的一系列标准品(从10^-14^到10^-9^，对应浓度为2到2×10^5^ Folate Unit)用于CTC的定量分析。所有的血液样本都是复孔检测，且有6个标准品，3个质控品(表 1)。

### 1.4 统计学分析

Folate Unit通过中位值及四分位数间距描述。两组之间CTC水平比较采用*Mann-Whitney U*检验分析，多组之间CTC水平比较采用*Kruskal-Wallis*检验。区别癌症与非癌症患者的最佳截域值(cutoff)是通过受试者工作特征曲线(receiver operating characteristic curve, ROC)分析得到，并计算得到每个指标的ROC曲线下面积(area under curve, AUC)。所有统计*P*值都是双侧检测，且统计分析采用SPSS 19.0 (SPSS Inc, Chicago, IL)或Prism 5.0(GraphPad Software Inc, San Diego, CA)处理。*P* < 0.05为差异有统计学意义。

## 2 结果

### 2.1 患者临床样本CTC水平

良性疾病和肺癌患者CTC中位值分别为8.08(4.19-11.99)、16.57(2.81-44.65)，肺癌患者CTC水平高于良性疾病患者(*P* < 0.001，图 2A)。

**2:**
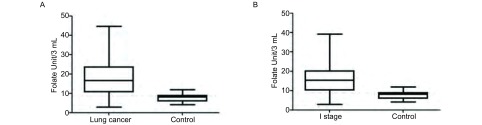
肺癌、Ⅰ期肺癌及肺部良性疾病患者CTC水平。A：肺部良性疾病患者、肺癌患者CTC水平；B：肺部良性疾病患者、Ⅰ期肺癌患者CTC水平。 The CTC level in lung cancer, Ⅰ stage of lung cancer and benign disease. A: The CTC level in lung cancer and benign disease; B: The CTC level in Ⅰ stage of lung cancer and benign disease.

我们通过分析Ⅰ期肺癌患者CTC的诊断价值来进一步评估通过FR检测CTC方法在早期诊断中应用的实用性和可能性。良性疾病患者和Ⅰ期肺癌患者CTC中位值分别为8.08(4.19-11.99)、15.39(2.81-39.09)(*P* < 0.001；图 2B)。

为探索肺癌患者CTC水平的意义，从受试者临床和病理特征(如确诊时患者的年龄(≤60岁*vs* > 60岁)、性别(男性*vs*女性)、吸烟史、肿瘤分级(G1 *vs* G2 *vs* G3)、TNM分期(Ⅰ期-Ⅱ期vs Ⅲ期-Ⅳ期)、病理亚型(ADC *vs* SCC)比较CTC的水平，结果如表 2所示，年龄、性别、吸烟史、肿瘤分级及病理亚型与CTC无关，但TNM分期中Ⅲ期-Ⅳ期患者CTC水平高于Ⅰ期及Ⅱ期患者CTC水平(*P* < 0.05)，其中Ⅱ期患者CTC水平略低于Ⅰ期患者可能与前者入组样本量较少有关。

**2:** 肺癌患者CTC水平 The CTC level of lung cancer

Characteristics	CTC Unit, Median (inter-quartile range)	*P*
Age (year)		
≤61	15.81 (2.81-39.09)	0.348
> 61	14.23 (2.88-46.37)	
Gender		
Male	14.38 (2.88-46.37)	0.766
Female	14.11 (2.81-39.09)	
Smoking status		
Current smoking	14.26 (2.88-46.37)	0.462
Non smoking	15.81 (2.81-39.09)	
Grade		
Poorly differentiated (G3)	11.40 (2.89-34.78)	0.103
Well/moderately differentiated (G1/G2)	15.52 (2.81-46.37)	
TNM stage		
Ⅰ	15.39 (2.81-39.09)	0.018
Ⅱ	14.38 (2.88-24.51)	
Ⅲ-Ⅳ	21.04 (4.52-44.65)	
Histopathologic subtype		
ADC	14.82 (2.81-46.37)	0.690
SCC	14.32 (2.89-44.65)	
TNM: tumor-node-metastasis.		

### 2.2 ROC分析

ROC曲线是用来确定CTC对NSCLC(ADC和SCC)患者的诊断有效性，CytoploRare^®^叶酸受体阳性循环肿瘤细胞检测试剂盒对肺癌诊断的cutoff值为8.70 Folate Unit/3 mL，灵敏度为82.5%，特异度为72.2%，AUC为0.849(95%CI: 0.777-0.922)。该方法对Ⅰ期肺癌患者的诊断灵敏度达到86.8%(33/38)。Ⅱ期、Ⅲ期、Ⅳ期肺癌患者阳性检出率分别为70.6%(12/17)、84.6%(22/26)、76.9%(10/13)。

### 2.3 与其他肿瘤标志物比较分析

我们通过比较CTC与目前临床上普遍使用的肺癌肿瘤标志物(NSE, CEA, CYFRA21-1)，进一步验证FR靶向PCR CTC检测方法对肺癌诊断的有效性。这些肿瘤标志物检测由Elecsys免疫分析仪(Roche Diagnostics, Bavaria, Germany)完成。

如图 3A，通过FR靶向PCR检测到的CTC较其他肿瘤标志物有更大的AUC(0.859; 95%CI: 0.779-0.939)，且更易去辨别患者是肺癌还是良性病，甚至该方法可以应用于早期的肺癌患者诊断，如图 3B，通过FR靶向PCR检测到的CTC对Ⅰ期肺癌患者相较其他肿瘤标志物有更大的AUC(0.912; 95%CI: 0.829-0.994)。

**3:**
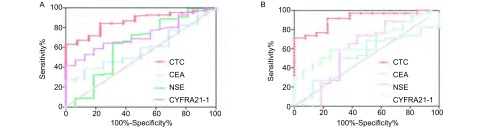
ROC分析比较CTC与肺癌一般肿瘤标志物对肺癌的诊断能力。A：比较CTC与肺癌一般肿瘤标志物曲线下面积(肺癌)；B：比较CTC与肺癌一般肿瘤标志物曲线下面积(Ⅰ期肺癌)。 The diagnostic efficiency of CTC and investigating clinical biomarkers in lung cancer. A: AUC of CTC and investigating clinical biomarkers in lung cancer; B: AUC of CTC and investigating clinical biomarkers in Ⅰ stage of lung cancer. AUC: area under curve.

### 2.4 术后监测

7例患者分别检测术前、术后2周内CTC水平，其中5例仍高于正常值，但其中仅1例在术后2周内CTC水平明显上升，其余均下降(图 4)，手术后即时检测CTC可发现术后升高患者，可能存在未被发现的隐匿病灶。

**4:**
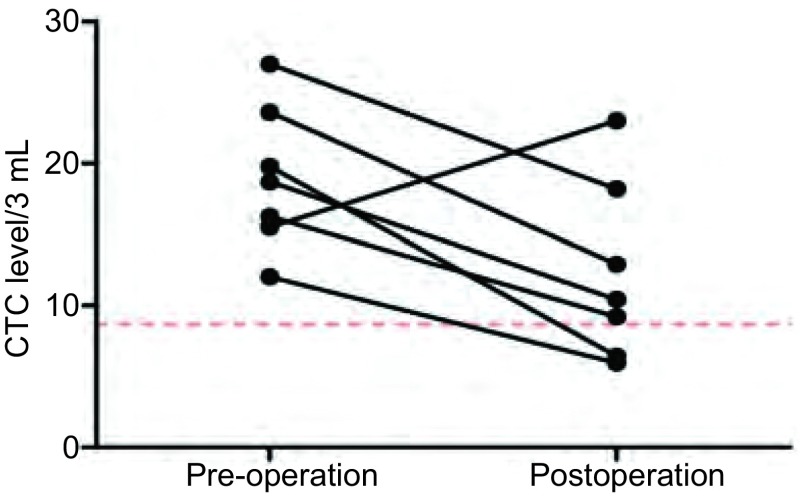
肺癌术后患者CTC水平较术前变化趋势图 The change of CTC level in lung cancer pre-operation and postoperation

## 3 讨论

NSCLC患者的CTC检测的敏感性和特异性仍然存在困难；尽管如此，这仍是一个需要发展的领域，目前还没有通用的检测方法适合所有类型的肿瘤^[12]^。CellSearch系统(Veridex, Raritan, NJ)是CTC的检测建立标准评估系统的先驱者，该系统已经获得了美国食品药品管理局批准作为晚期乳腺癌、前列腺癌和结肠癌CTC的检测^[19]^。该技术是依赖上皮细胞标记物来识别CTC，然而，在肺癌EMT中丢失这些标记物而致使该检测方法无效^[12, 20]^。另外，同理也很难通过EpCAM微流体CTC芯片检测到NSCLC患者循环肿瘤细胞。另外可以通过上皮肿瘤细胞大小分离检测CTC，该技术基于细胞的形态学特征，而不利用细胞表面免疫标记物。之前有研究发现在肺癌患者中，通过上皮肿瘤细胞大小分离法检测CTC的阳性率要高于CellSearch检测系统^[21]^。然而，捕获的细胞缺少后续的常规细胞学验证，假阳性率高需要采用免疫表型特征学去改进此方法^[22]^。

FR是一种细胞表面受体糖蛋白，它主要是在卵巢癌和肺癌肿瘤中高表达，目前是NSCLC患者药物治疗的一个潜在的重要靶点^[23]^。一项研究^[24]^表明在大约75.7% NSCLC患者中FR是过量表达的，而且在肺腺癌中的表达水平更高于肺鳞癌。尽管一些正常组织包括肺部正常组织也能表达FR，但在血液中除了活性巨噬细胞亚群外其他没有非正常细胞能够表达功能性FR。然而这些活性巨噬细胞在健康志愿者或者肺部良性疾病患者中很难被检测到。在本研究中，我们将FR靶向PCR CTC检测方法应用于肺癌，发现肺部良性疾病患者中循环肿瘤细胞的水平低于肺癌组(*P* < 0.001)。FR靶向PCR法是我们CTC检测方法中另一个核心技术，该方法通过负向筛选CTC，后经叶酸配体-寡核苷酸标记富集的CTC，最后通过RT靶向PCR定量分析。Parker等^[14]^通过使用定量放射配体结合分析法得出NSCLC样本每毫克溶解膜蛋白表达的FR平均水平为6.11 pmol，这意味着在一个NSCLC细胞表面大约有多余10万的FR。在NSCLC患者外周血中每一个循环肿瘤细胞就能增加10万个数量级的连接叶酸的寡核苷酸和叶酸受体。通过这两级放大，可以检测到3 mL血液样本中极少量的CTC。

循环中检测出CTC并不一定意味着患者已经存在远处转移。肿瘤转移是个相对复杂的过程。CTC侵袭入原发灶以外的第二器官(如骨髓等)，可称之为DTC(disseminated tumor cells)^[25]^。一般而言，具有干细胞特征的DTC聚集成团，并且形成微转移灶，同时该转移灶可以逃避免疫系统的识别，肿瘤的远处转移才可能发生^[26, 27]^。在肿瘤的浸润和转移过程中，肿瘤细胞会表现出更多的间叶表型特征，失去正常情况下细胞间的相互作用，即存在EMT过程，从而使其更容易侵入血管内皮而进入血循环^[28]^。在EMT过程中，肿瘤细胞下调了上皮组织特定标记物如CK和上皮细胞粘附分子(EpCAM)的表达，上调了间叶组织标记物如波形蛋白的表达^[29, 30]^。但是，EMT并不是一个“全或无”的过程，很多CTC可以同时表达间叶组织标记物和上皮组织。这对现行的一些CTC的检测方法有着重要的影响。例如针对上皮组织标记物的检测方法就无法检测到发生EMT的CTC。

另外一个重要的问题是，早期肿瘤外周循环中是否可以检测到CTC，也就是说CTC迁移入血的过程是发生在肿瘤早期还是晚期？理论上讲，肿瘤大小超过2 mm左右时便可诱导血管生成进入肿瘤，提供血供，从而为肿瘤细胞迁移入血提供基础。Husemann等^[31]^通过研究证实了，在转基因小鼠种植后约17周-18周，循环中即可检出CK与HER2同时阳性表达的细胞，而此时原发肿瘤体积还小于1 mm3。另有研究的结果也再次印证了上述结果^[32, 33]^，其中He等^[33]^将表达中等量FR的M109鼠肺癌细胞通过皮下注射的方式被移植到BALB/c小鼠的背部侧面。移植2周后，在小鼠耳部的血管中每分钟可以检测到大约1.4个CTC细胞。而到了移植后的第3周和第4周，则每分钟可分别检测7个和18个CTC。随着肿瘤逐渐增大，CTC的数量呈指数级别上升。在肿瘤移植的前4周内，没有在任何组织切片中发现存在转移性癌症的迹象。因此，理论上而言，在肿瘤发生发展的早期，循环中即可检出CTC。最近有研究表明，胰腺癌模型在肿瘤进展的早期阶段，甚至在恶化之前就能够检测到CTC，这意味着CTC可以作为癌症的早期诊断的一个潜在的生物标志物。我们的研究结果同样表明，通过FR靶向PCR检测CTC的方法在Ⅰ期肺癌患者中检测灵敏度达到86.8%。在我们研究中的受试者同样需要接受肺癌肿瘤标志物的检测，如NSE、CEA、CYFRA21-1，比较他们与CTC的诊断能力。与这些肿瘤标志物相比，FR靶向PCR CTC检测方法具有最高的AUC。

本研究发现肺癌患者术后两周内检测CTC水平明显升高，既往文献也发现，手术中或术后即时检测CTC，会发现CTC水平的升高，可能原因：手术挤压导致肿瘤细胞入血(非转移性)或者肺部创伤，导致大量肺上皮细胞入血。在术后一个月检测CTC如果水平升高，可能存在未被发现的隐匿病灶。我们研究中入组的部分样本在持续随访中。

总之，FR阳性CTC可以作为肺癌有效的生物诊断标记物，甚至应用于早期肿瘤诊断。进一步探索肺癌患者术前CTC水平和预后的相关性及术后的实时监测复发是必要的。

## References

[1] Xie L, Ugnat AM, Morriss J (2003). Histology-related variation in the treatment and survival of patients with lung carcinoma in Canada. Lung Cancer.

[2] Takita H, Pitoniak RF (2000). Induction chemtherapy for locoregional lung cancer using paclitaxel combination. A preliminary report. J Exp Clin Cancer Res.

[3] Pantel K, von Knebel Doeberitz M, Izbicki JR (1997). Disseminierte tumorzellen: diagnostik, prognostische relevanz, phanotypisierung und therapeutische strategien. Chirurg.

[4] Klein CA (2009). Parallel progression of primary tumours and metastases. Nat Rev Cancer.

[5] Lou J, Ben S, Yang G (2013). Quantification of rare circulating tumor cells in non-small cell lung cancer by ligand-targeted PCR. PLoS One.

[6] Yu Y, Chen Z, Dong J (2013). Receptor-positive circulating tumor cellsas a novel diagnostic biomarker in non-small cell lung cancer. Transl Oncol.

[7] Chen X, Zhou F, Li X (2015). Folate receptor-positive circulating tumor cell detected by LT-PCR based method as a diagnostic biomarker for non-small cell lung cancer. J Thorac Oncol.

[8] Sawabata N, Okumura M, Utsumi T (2007). Circulating tumor cells in peripheral blood caused by surgical manipulation of non-small-cell lung cancer: pilot study using an immunocytology method. Gen Thorac Cardiovasc Surg.

[9] Rolle A, Günzel R, Pachmann U (2005). Increase in number of circulating disseminated epithelial cells after surgery for non-small cell lung cancer monitored by MAINTRAC? is a predictor for relapse: a preliminary report. World J Surg Oncol.

[10] Muinelo-Romay L, Vieito M, Abalo A (2014). Evaluation of circulating tumor cells and related events as prognostic factors and surrogate biomarkers in advanced NSCLC patients receiving first-line systemic treatment. Cancers.

[11] Gorges TM, Tinhofer I, Drosch M (2012). Circulating tumour cells escape from EpCAM-based detection due to epithelial-to-mesenchymal transition. BMC Cancer.

[12] Young R, Pailler E, Billiot F (2012). Circulating tumor cells in lung cancer. Acta Cytol.

[13] Krebs MG, Sloane R, Priest L (2011). Evaluation and prognostic significance of circulating tumor cells in patients with non-small-cell lung cancer. J Clin Oncol.

[14] Parker N, Turk MJ, Westrick E (2005). Folate receptor expression in carcinomas and normal tissues determined by a quantitative radioligand binding assay. Anal Biochem.

[b15] 15Lou J, Zhou C, Wu J, et al. A multicenter clinical trial of lung cancer circulating tumor cell assay with the largest sample size (1210 cases) in China. 2016 AACR Annual Meeting-abstra: 2247.

[16] Caceres G, Puskas JA, Magliocco AM (2015). Circulating tumor cells: a window into tumor development and therapeutic effectiveness. Cancer Control.

[17] Xiao F, Jiang M, Du D (2013). Orexin A regulates cardiovascular responses in stress-induced hypertensive rats. Neuropharmacology.

[18] Jiang M, Wang Q, Karasawa T (2014). Sodium-glucose transporter-2 (SGLT2; SLC5A2) enhances cellular uptake of aminoglycosides. PLoS One.

[19] Lucci A, Hall CS, Lodhi AK (2012). Circulating tumor cells in non-metastatic breast cancer: a prospective study. Lancet Oncol.

[20] Lecharpentier A, Vielh P, Perez-Moreno P (2011). Detection of circulating tumour cells with a hybrid (epithelial/mesenchymal) phenotype in patients with metastatic non-small cell lung cancer. Br J Cancer.

[21] Hofman V, Ilie MI, Long E (2011). Detection of circulating tumor cells as a prognostic factor in patients undergoing radical surgery for non-small-cell lung carcinoma: comparison of the efficacy of the CellSearch AssayTM and the isolation by size of epithelial tumor cell method. Int J Cancer.

[22] Hofman VJ, Ilie MI, Bonnetaud C (2011). Cytopathologic detection of circulating tumor cells using the isolation by size of epithelial tumor cell method:promises and pitfalls. Am J Clin Pathol.

[23] Thomas A, Maltzman J, Hassan R (2013). Farletuzumab in lung cancer. Lung Cancer.

[24] Nunez MI, Behrens C, Woods DM (2012). High expression of folate receptor alpha in lung cancer correlates with adenocarcinoma histology and EGFR. J Thorac Oncol.

[25] Haber DA, Velculescu VE (2014). Blood-based analyses of cancer: circulating tumor cells an circulating tumor DNA. Cancer Discov.

[26] Pantel K, Alix-Panabieres C, Riethdorf S (2009). Cancer micrometastases. Nat Rev Clin Oncol.

[27] Aguirre-Ghiso JA (2007). Models, mechanisms and clinical evidence for cancer dormancy. Nat Rev Cancer.

[28] Barrière G, Tartary M, Rigaud M (2012). Epithelial mesenchymal transition:a new insight into the detection of circulating tumor cells. ISRN Oncol.

[29] Matrone MA, Whipple RA, Balzer EM (2010). Microtentacles tip the balance of cytoskeletal forces in circulating tumor cells. Cancer Res.

[30] Hou JM, Krebs M, Ward T (2011). Circulating tumor cells as a window on metastasis biology in lung cancer. Am J Pathol.

[31] Husemann Y, Geigl JB, Schubert F (2008). Systemic spread is an early step in breast cancer. Cancer Cell.

[32] Eyles J, Puaux AL, Wang X (2010). Tumor cells disseminate early, but immunosurveillance limits metastaticout growth, in a mouse model of melanoma. J Clin Invest.

[33] He W, Wang H, Hartmann LC (2007). *In vivo* quantitation of rare circulating tumor cells by multiphoton intravital flow cytometry. Proc Natl Acad Sci U S A.

